# Supplementary material for: Selection of GalNAc-conjugated siRNAs with limited off-target-driven rat hepatotoxicity
Source: Nat Commun. 2018 Feb 19;9:723. doi: 10.1038/s41467-018-02989-4 (PMC5818625; doi:10.1038/s41467-018-02989-4)
Supplement: Supplementary file 1 — Supplementary Information [file 41467_2018_2989_MOESM1_ESM.docx]

**
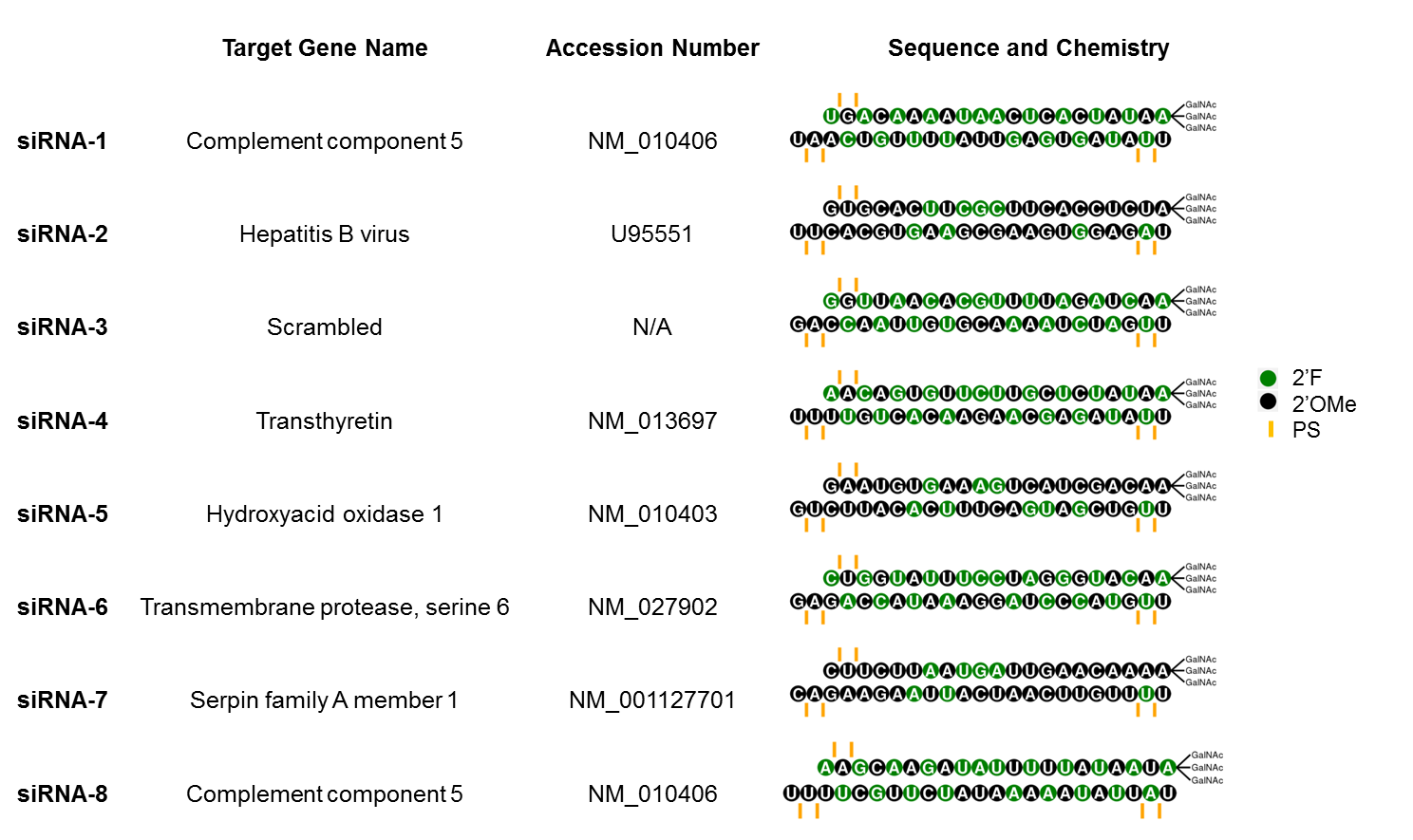
**

**Supplementary Figure 1. Toolkit GalNAc-siRNAs used in these studies.** Abbreviations are as follows: 2′F, 2′- deoxy-2′-fluoro; 2′OMe, 2′-*O*-methyl; PS, phosphorothioate; GalNAc, *N*-acetylgalactosamine.

**
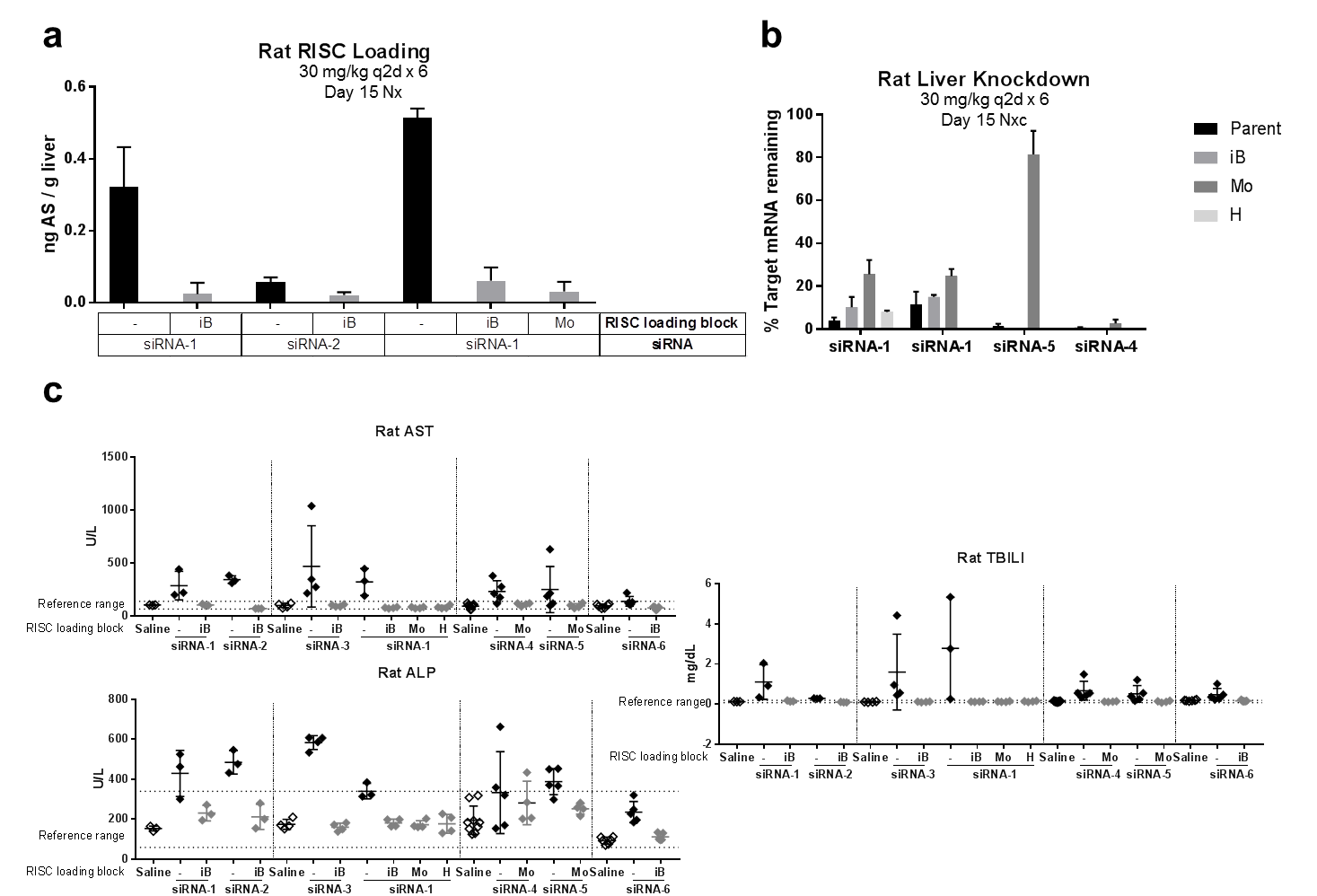
**

**Supplementary Figure 2. Effects of antisense strand 5’-modifications on activity and rat hepatotoxicity . (a)** Liver RISC loading of GalNAc-siRNAs with or without 5’-caps as assessed at necropsy (nx) by stem-loop RT-qPCR for the antisense strand (AS). **(b)** Liver mRNA knockdown with or without 5’-caps as assessed at necropsy by RT-qPCR for target mRNA and normalized to a housekeeping mRNA (18S rRNA), relative to the saline control group. **(c)** Serum aspartate aminotransferase (AST), alkaline phosphatase (ALP) and total bilirubin (TBILI) levels measured at necropsy for the RISC loading block studies Error bars represent standard deviation of the mean. N = 3 males (6-8 weeks old) per group. Q2d, every other day dosing; iB, inverted abasic; Mo, morpholino; H, 5’-deoxy.

**
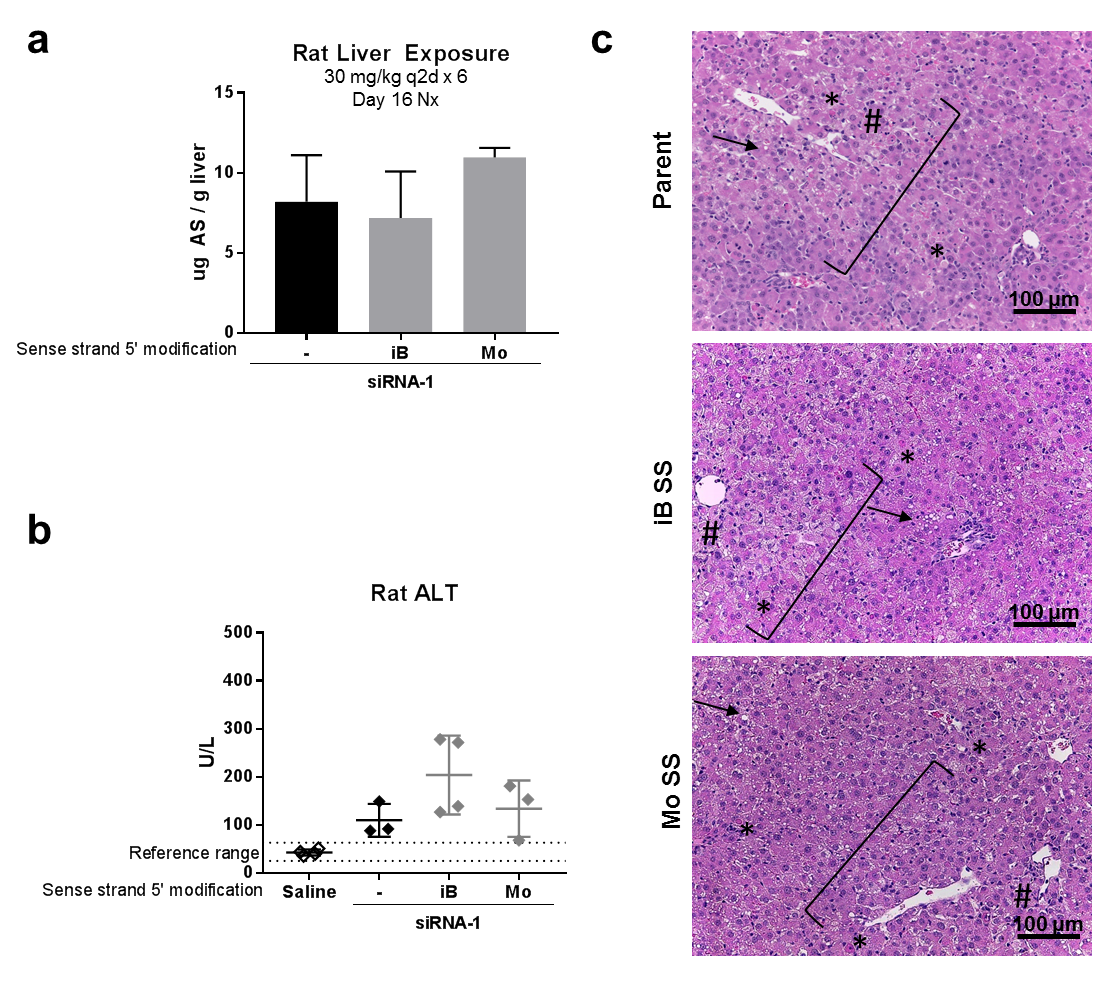
**

**Supplementary Figure 3. Effects of sense strand 5’-modifications on rat hepatotoxicity. (a)** Liver exposures for a toxic GalNAc-siRNA with or without modifications on the 5’-end of the sense strand (SS) in rat toxicity studies as assessed at necropsy (nx) by stem-loop RT-qPCR for the antisense strand (AS). **(b)** Serum alanine aminotransferase (ALT) levels measured at necropsy. Error bars represent standard deviation of the mean. **(c)** H&E staining of liver sections collected at necropsy. The toxic siRNA had microscopic findings consisting of hepatocellular degeneration (bracket), single cell necrosis (*), increased sinusoidal cells consistent with Kupffer cell hyperplasia and/or infiltrating leukocytes (#), and hepatocellular vacuolation (arrow). The addition of sense strand caps had no effect on the incidence or severity of findings. All microscopic liver findings are tabulated in Supplementary Table 6. N = 3 males (6-8 weeks old) per group. Q2d, every other day dosing; iB, inverted abasic; Mo, morpholino.


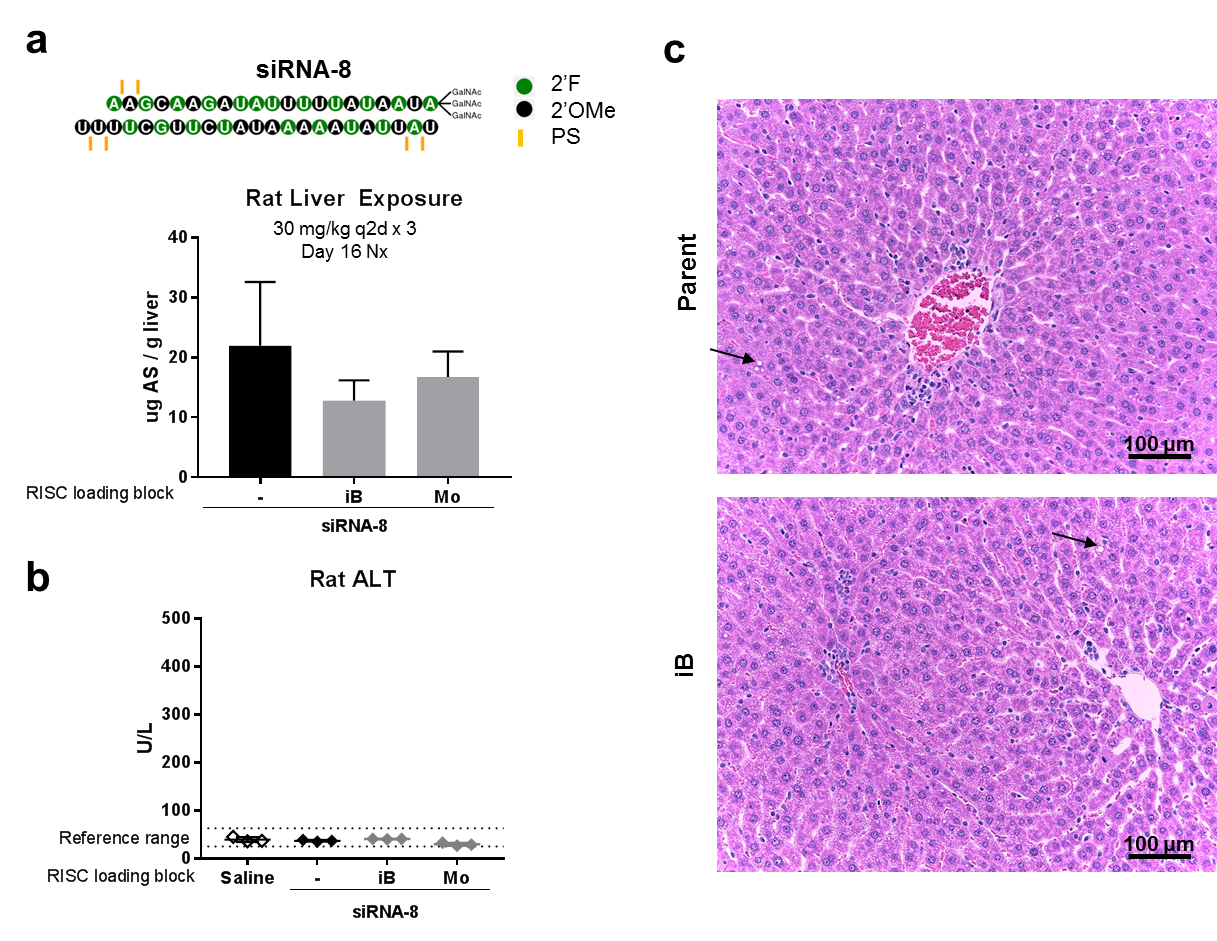


**Supplementary Figure 4. Effects of 5’-modifications on a non-toxic GalNAc-siRNA.** **(a)** Liver exposures for a non-toxic GalNAc-siRNA with or without modifications on the 5’-end of both sense strand and the antisense strands in rat toxicity studies as assessed at necropsy (nx) by stem-loop RT-qPCR for the antisense strand (AS). **(b)** Serum alanine aminotransferase (ALT) levels measured at necropsy. Error bars represent standard deviation of the mean. **(c)** H&E staining of liver sections collected at necropsy. Administration of the known non-toxic siRNA with or without 5’-caps led to minimal hepatocellular vacuolation (arrow) in both cases. Microscopic liver findings are tabulated in Supplementary Table 7. N = 3 males (6-8 weeks old) per group. Q2d, every other day dosing; iB, inverted abasic; Mo, morpholino.

**
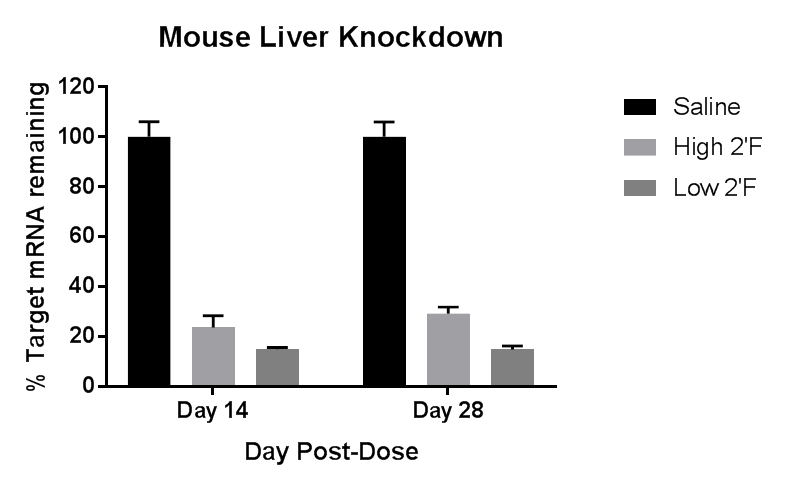
**

**Supplementary Figure 5. Impact of 2′F content on *in vivo* potency.** After a single subcutaneous injection of 3 mg/kg of a high 2′F version (48% 2′F and 52% 2′OMe) and a low 2′F version (21% 2′F and 79% 2′OMe) of siRNA-6 to C57BL/6 female mice, liver on-target mRNA knockdown was assessed on Days 14 and 28 by RT-qPCR for target mRNA and normalized to a housekeeping mRNA (GAPDH), relative to the saline control group. Error bars represent standard deviation of the mean. N = 3 males (6-8 weeks old) per group.

**
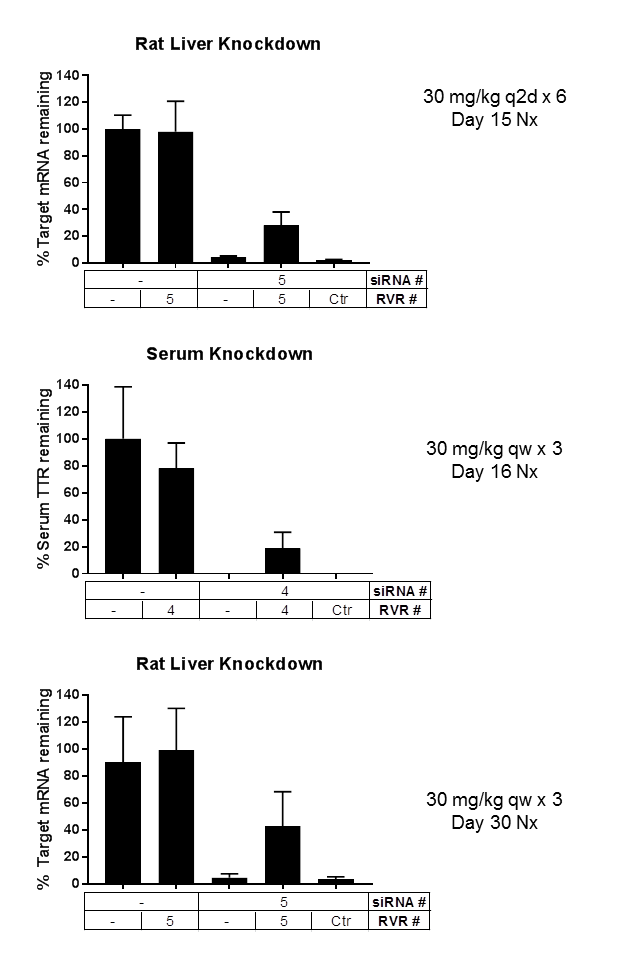
**

**Supplementary Figure 6. Effects of REVERSIR compounds on RNAi activity.** Liver on-target mRNA knockdown with siRNA-1 and siRNA-5 was assessed at necropsy (nx) by RT-qPCR for target mRNA and normalized to a housekeeping mRNA (18S rRNA), relative to the saline control group. On-target serum protein levels with siRNA-4 were assessed at necropsy by ELISA, relative to the saline control group. Error bars represent standard deviation of the mean. N = 3 males (6-8 weeks old) per group. Q2d, every other day dosing; qw, weekly dosing.


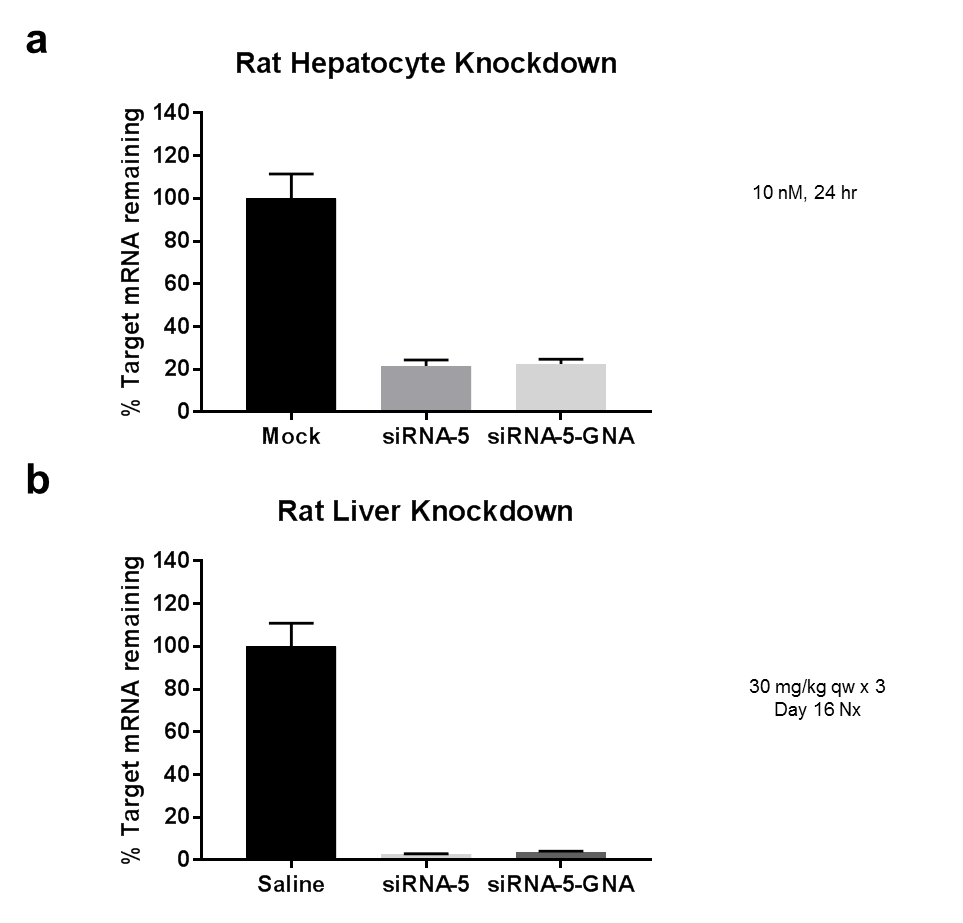


**Supplementary Figure 7. Impact of seed-pairing destabilization using GNA on activity. (a)** Rat hepatocyte mRNA knockdown was assessed at 24 hrs post-10 nM transfection by reverse transcription-quantitative PCR (RT-qPCR) for target mRNA and normalized to a housekeeping mRNA (18S rRNA), relative to the mock transfection. **(b)** Liver mRNA knockdown was assessed at necropsy by RT-qPCR for target mRNA and normalized to a housekeeping mRNA (18S rRNA), relative to the saline control group. Error bars represent standard deviation of the mean. N = 3 males (6-8 weeks old) per group. Qw, weekly dosing; GNA, glycol nucleic acid.

**Supplementary Table 1. Blocking RISC loading mitigates hepatotoxicity.** Histologic findings with RNAi-active and RNAi-inactive GalNAc-siRNAs. The range of severity grade for each histologic finding is indicated on a scale of 1-5 with 1 indicating minimal severity and 5 indicating severe severity.

**Supplementary Table 2. Effects of sense strand 5’-modifications on rat hepatotoxicity.** Histologic findings with a GalNAc-siRNA with 5’-RISC blocking modifications on the sense strand. The range of severity grade for each histologic finding is indicated on a scale of 1-5 with 1 indicating minimal severity and 5 indicating severe severity. iB, inverted abasic; Mo, morpholino.

**Supplementary Table 3. Impact of 5’-modification on hepatotoxicity of a non-toxic siRNA.** Histologic findings with a non-toxic GalNAc-siRNA with 5’-RISC blocking modifications on both the sense and antisense strand. The range of severity grade for each histologic finding is indicated on a scale of 1-5 with 1 indicating minimal severity and 5 indicating severe severity. iB, inverted abasic; Mo, morpholino.

**Supplementary Table 4. Impact of 2’-chemical modifications on hepatotoxicity.** Histologic findings with GalNAc-siRNAs with high or low 2′F content. The range of severity grade for each histologic finding is indicated on a scale of 1-5 with 1 indicating minimal severity and 5 indicating severe severity.

**Supplementary Table 5. Blocking RNAi activity mitigates hepatotoxicity.** Histologic findings with GalNAc-siRNAs with or without treatment with REVERSIR^TM^ compounds targeting the antisense strand. The range of severity grade for each histologic finding is indicated on a scale of 1-5 with 1 indicating minimal severity and 5 indicating severe severity.

**Supplementary Table 6. Swapping seed regions mitigates hepatotoxicity.** Histologic findings with GalNAc-siRNAs with or without seed region swapping. The range of severity grade for each histologic finding is indicated on a scale of 1-5 with 1 indicating minimal severity and 5 indicating severe severity.

**Supplementary Table 7. Destabilizing seed binding to off-targets mitigates toxicity.** Histologic findings with parent and seed GNA-modified GalNAc-siRNA. The range of severity grade for each histologic finding is indicated on a scale of 1-5 with 1 indicating minimal severity and 5 indicating severe severity. GNA, glycol nucleic acid.
